# Novel advances on pathophysiological mechanisms, clinical manifestations, and treatment of antiphospholipid syndrome

**DOI:** 10.3389/fimmu.2025.1639065

**Published:** 2025-08-21

**Authors:** Qing-Nan Zhu, Xiang-Bo Qi, Shu-Wei Ren, Yu-Ye Li, Ze-Wen Yan, Yu Sun, Yan Shi, Qing-Si Wen, Mao-Mao Wu, Da-Peng Wang

**Affiliations:** ^1^ Department of Nephrology, The First Affiliated Hospital, Dalian Medical University, Dalian, China; ^2^ Institute of Integrative Medicine, Dalian Medical University, Dalian, China

**Keywords:** antiphospholipid syndrome, systemic lupus erythematosus, thromboinflammation, thrombosis, anticoagulants

## Abstract

Antiphospholipid antibody syndrome (APS) is an autoimmune disorder characterized by arterial and venous thrombosis, pregnancy-related complications, and persistent antiphospholipid antibodies. These manifestations pose significant risks to patient health and reproductive outcomes. Initially regarded as a manifestation of systemic lupus erythematosus (SLE), APS exhibits a close epidemiological association with SLE, occurring at significantly higher incidence in SLE patients. The precise pathophysiological relationship between these diseases remains unclear. Nevertheless, as an independent clinical disease, research on APS pathological mechanisms continues to advance comprehensively. The publication of the “2023 ACR/EULAR antiphospholipid syndrome classification criteria” provides refined diagnostic standards. Consequently, this review synthesizes prior studies to clarify APS pathophysiological mechanisms, explore its relationship with SLE, update emerging treatments, and provide insights for clinical management.

## 1 Introduction

SLE is a chronic systemic autoimmune disease characterized by anti-dsDNA antibodies and immune-mediated inflammatory tissue damage. Principal clinical manifestations include malar rash, arthritis, lupus nephritis, and neuropsychiatric symptoms, with higher prevalence in women of childbearing age (1). APS is an autoimmune disease characterized by persistent aPL, and clinical manifestations include venous and arterial thrombosis, microvascular thrombosis, obstetric complications, cardiac valve disease, and thrombocytopenia. Laboratory diagnostic criteria include positivity for lupus anticoagulant (LA), IgG/IgM anticardiolipin antibodies (aCL), and/or IgG/IgM anti-β_2_ glycoprotein I (anti-β_2_GPI) antibodies (2). Notably, more than 40% of SLE patients demonstrate aPL positivity, and a significant proportion of primary APS patients exhibit anti-nucleosome antibodies (3). Notably, APS patients carry an 80.7-fold increased risk of developing SLE compared with non-APS individuals. Concurrent SLE-APS is associated with autoimmune hemolytic anemia, glomerular thrombosis, myocardial infarction, and elevated mortality (4); however, the pathophysiological interaction between these two diseases remains incompletely understood.

This article reviews the pathogenesis, pathophysiological interactions, and treatment for SLE and APS.

## 2 Pathophysiological mechanisms

SLE and APS are distinct autoimmune disorders with significant pathophysiological overlap. They frequently co-occur in the same patient, and APS may develop secondary to SLE. Based on the core pathophysiological mechanisms of APS, its pathogenesis primarily involves pathogenic antiphospholipid antibodies, which trigger B-cell activation and elevated circulating immune complexes. These antibodies further drive complement activation as well as neutrophil extracellular trap (NET) formation. Additionally, they induce mitochondrial dysfunction and type I interferon hyperreactivity. Crucially, these antibodies promote a pro-thrombotic state through increased tissue factor (TF) expression, disruption of the annexin V protective shield on cell surfaces, and impaired fibrinolytic pathway function, ultimately leading to thrombosis and obstetric complications (5).

aPLs primarily activate endothelial cells (ECs), platelets, monocytes, and neutrophils through interactions with phospholipid-binding proteins, phospholipids (PL), or their complexes. This initiates the coagulation cascade and promotes thrombosis. Under physiological conditions, ECs maintain blood flow by releasing nitric oxide (NO) and prostaglandin I2 (PGI_2_), which inhibit platelet aggregation and clotting factor activation. Additionally, ECs express tissue factor pathway inhibitors (TFPI) and thrombomodulin (TM) and produce urokinase-type plasminogen activators (u-PA) and tissue plasminogen activators (t-PA) to promote fibrinolysis. aPL activation suppresses these protective molecules while upregulating tissue factor (TF) and cell adhesion molecules, enhancing leukocyte and platelet adhesion (6–8).

**Figure 1:**
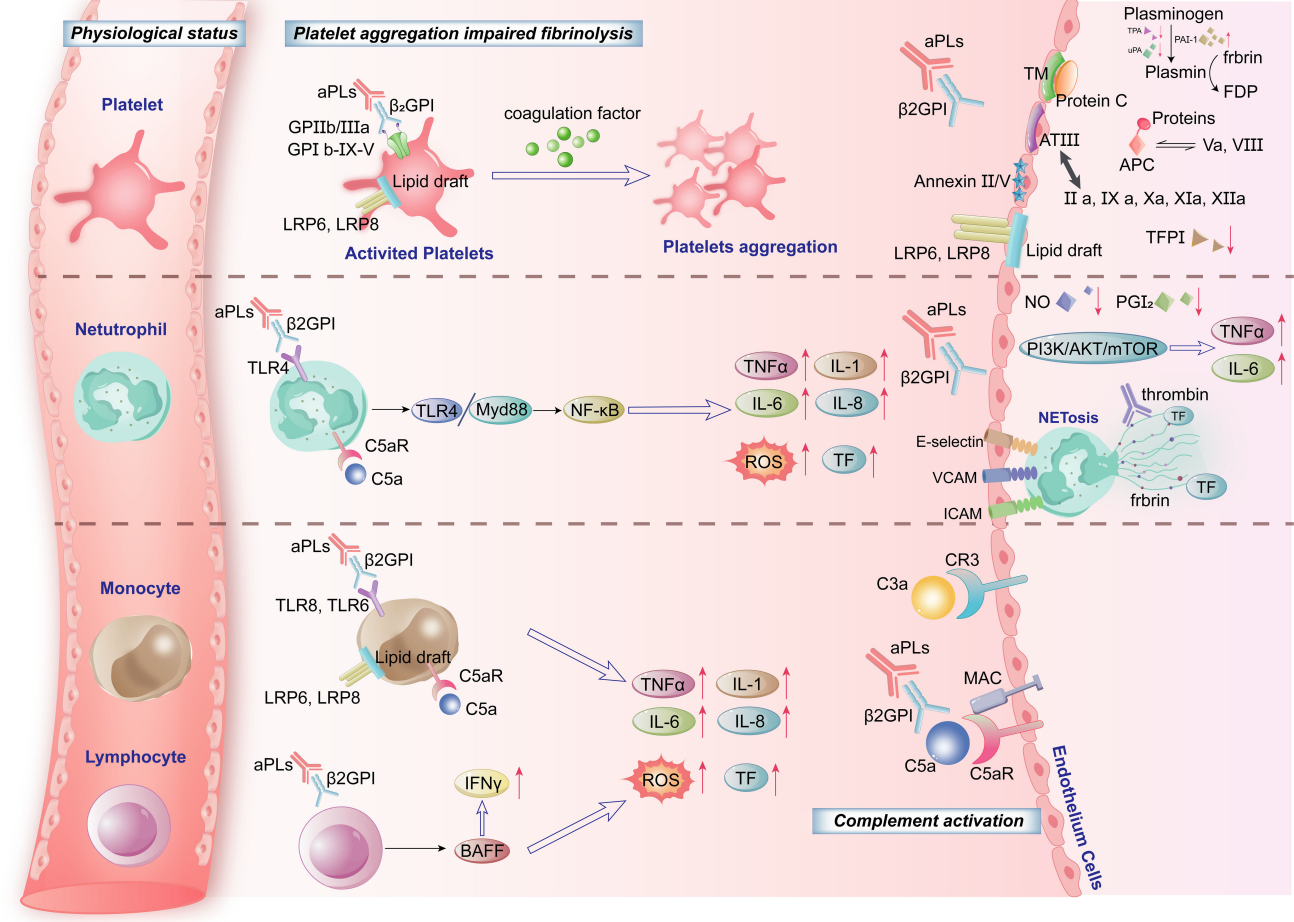
Pathogenesis of antiphospholipid syndrome (APS): cellular mechanisms.

Activated monocytes and neutrophils are activated to release inflammatory cytokines, including tumor necrosis factor (TNF-α), interleukin-1β (IL-1β), and interleukin-6 (IL-6). These cytokines further activate platelets and ECs, thereby exacerbating thrombosis and promoting pro-inflammatory responses (9). These processes ultimately contribute to microvascular thrombosis, inflammation, and end-organ damage in vascular, obstetric, neurological, and cardiovascular systems (summarized in **Figure 1**).

### 2.1 Anti-β_2_ glycoprotein I antibody, lupus anticoagulant, anti-cardiolipin

β_2_-Glycoprotein-I (β_2_GPI) is a 50-kDa plasma glycoprotein also termed apolipoprotein H (ApoH). β_2_GPI consists of five short consensus repeats, called “sushi” domains. Its C-terminal domain V binds negatively charged cell surface receptors (10). β_2_GPI circulates in three conformations: a circular plasma conformation, S-shaped, and open fishhook. The cryptic epitope on the N-terminal domain I serves as the immunodominant target for pathogenic IgG/IgM anti-β_2_GPI antibodies. This cationic phospholipid-binding glycoprotein is secreted by hepatocytes, monocytes, trophoblasts, ECs, and platelets. Under oxidative stress, positively charged residues and hydrophobic regions engage phospholipid membranes, disrupting circular conformation and exposing the domain I epitope of β_2_GPI (10, 11).

Anti-β_2_GPI antibodies promote a prothrombotic state by inhibiting natural anticoagulants and fibrinolytic pathways and by activating monocytes, neutrophils, platelets, and vascular ECs. APS patients show altered T-cell reactivity to β_2_GPI, possibly due to neoepitopes formed by protein structural changes induced by chronic inflammation and oxidative stress. Oxidized and glucose-modified β_2_GPI activate immature monocyte-derived dendritic cells, enhance allostimulatory capacity in mixed lymphocyte reaction, and modulate dendritic cell activation (12). Additionally, aPLs stimulate the expression of TNFα in monocytes through activation of the Toll-like receptor TLR4, TLR7, and TLR8 pathways and induce the release of TF from the surface of monocytes and ECs (13). TLR4 facilitates anti-β_2_GPI/β_2_GPI complex binding to monocytes and ECs, activating p38 MAPK, MEK-1/ERK, and NF-κB via TLR4/MyD88 and TLR4/TRIF pathways. NF-κB activation upregulates the tissue factor (TF) and increases the expression of vascular cell adhesion molecule-1 (VCAM-1), intercellular adhesion molecule (ICAM), and E-selectin (14). Molecular simulations demonstrate that IgG anti-β_2_GPI binding to TLR4 induces IRAK phosphorylation, NF-κB nuclear translocation, enhanced EC VCAM-1 expression, and monocyte TNF-α release. Inhibitors of TLR4, p38 MAPK, or NF-κB significantly reduce TNF-α, IL-1β, and IL-6 mRNA in anti-β_2_GPI antibody-stimulated macrophages (14). Anti-β_2_GPI antibodies bind and downregulate thrombin activity. These antibodies also adhere to platelets, enhance platelet and endothelial cell activation, and initiate thrombotic and inflammatory cascades. Consequently, they activate proatherogenic pathways and induce vascular cell dysfunction, directly contributing to thrombotic pathogenesis (15). Genetic engineering-mediated β_2_GPI-deficient murine models revealed that β_2_GPI potentiates platelet activation and thrombus formation through facilitating PAR3-dependent thrombin signaling transduction. Experimental data demonstrated that β_2_GPI deficiency resulted in attenuated thrombosis across multiple *in vivo* models concomitant with prolonged bleeding time, indicating β_2_GPI’s dual regulatory roles in both physiological hemostasis and pathological thrombogenesis (16). The anti-β_2_GPI antibody significantly increased thrombus size after laser-induced arterial injury in a mouse model (17). The 13th International Antiphospholipid Antibody Congress proposed that the IgA β_2_GPI test could be considered for APS patients with typical negative IgG and IgM (18). A European cohort study of 137 primary APS patients found that high-affinity anti-β_2_GPI antibodies were linked to venous thrombosis occurrence, whereas low-affinity antibodies predominated in non-APS, especially those with SLE (19).

aCL and anti-β_2_GPI antibodies induce TF expression in monocytes and ECs, contributing to the prothrombotic state of APS. LA confers a higher thrombotic risk than aCL by forming bivalent complexes with increased phospholipid affinity, competing with coagulation factors (20). Diluted Russell viper venom time (dRVVT) and LA-sensitive activated partial thromboplastin time (aPTT) are often used in clinical practice. LA is the primary predictor of adverse pregnancy outcomes related to aPL beyond 12 weeks of pregnancy (21). Among aPLs, aCL demonstrates higher sensitivity, whereas anti-β_2_GPI antibody exhibits superior specificity (2). Triple antibody testing provides greater diagnostic accuracy than single assays, enabling improved APS diagnosis and risk stratification (22).

In a consecutive cohort of 175 patients with suspected APS, approximately one-third of seronegative APS (SN-APS) cases exhibited reactivity to ≥1 non-standard antibody (23). The combination of non-standard aPLs improved diagnostic accuracy. Other APS antigen targets may include non-standard APS components such as IgA aCL and anti-β_2_GPI antibodies, antibodies against phospholipid or phospholipid/protein antigens such as anti-phosphatidylserine/prothrombin (aPS/PT), PS, activated protein C (APC), and Annexin II/V resistance (24). IgA aCL and anti-β_2_GPI antibodies provide supplementary diagnostic value in APS. *In vivo* studies in mice demonstrated that IgA anti-β_2_GPI antibodies induced larger thrombi and elevated TF and were significantly associated with arterial thrombosis (25).

Anti-prothrombin antibodies (aPT) targeting phosphatidylserine/prothrombin (aPS/PT) complexes represent unclassified biomarkers. Clinical studies demonstrate that persistent aPS/PT antibody positivity, thrombosis events, triple positivity for aPS/PT IgG/IgM antibodies, and LA activity significantly correlate with higher Global Antiphospholipid Syndrome Scores (GAPSS) (23). Annexin II and V have high affinity for phospholipids and play a key role in the regulation of the coagulation cascade (26). Annexin V is a glycoprotein abundant in vascular ECs, binds negatively charged phospholipids, and forms protective crystals on phosphatidylserine surfaces. These crystals block phospholipid-dependent thrombin binding sites, thereby inhibiting procoagulant/proinflammatory activities and regulating apoptosis (27). In the placenta, Annexin V is expressed by syncytiotrophoblasts and plays a key role in maintaining placental integrity and regulating thrombosis. IgG anti-ANXA5 antibodies were associated with arterial thrombotic events in 170 APS patients and 104 non-APS patients (OR 2.60; 95% CI, 1.44–4.71) and with venous thrombosis events (OR, 2.80; 95% CI, 1.55–5.06); thus, IgG anti-ANXA5 antibodies can be used as predictive biomarkers to identify patients at high risk of thrombosis (28–30). Prothrombin is also one of the target antigens of aPLs. Annexin II (A2) is a fibrinolytic receptor on the surface of ECs, which binds tissue plasminogen activator (tPA) and β_2_GPI, serving as a high-affinity receptor for anti-β_2_GPI antibody complexes. The serum anti-A2 antibodies were significantly elevated in 434 APS patients versus healthy controls (*P* = 0.017), non-autoimmune thrombosis patients, and non-thrombotic lupus patients (*P* = 0.001) (31). A2 antibodies increased cerebral infarct volume (133.9 ± 3.3 mm³ vs. 113.7 ± 7.4 mm³; P = 0.017) and worsened neurological outcomes (2.2 ± 0.2 vs. 1.5 ± 0.18; P = 0.03) in murine stroke models, establishing A2 as an independent cerebral thrombosis risk factor (32, 33).

### 2.2 NETosis

Neutrophil extracellular traps (NETs) are extracellular structures composed of chromatin, DNA, histones, and granular proteins released during NETosis. These structures trap pathogens and exhibit pro-inflammatory and pro-thrombotic properties, contributing to malignancies, sepsis, SLE, and APS (34).

In SLE and APS, enhanced NET release and impaired clearance of NET components lead to autoantigen exposure, activating autoreactive B cells and triggering autoantibody production, whereas NET-associated cytokines stimulate plasmacytoid dendritic cells to secrete IFNα. Additionally, the presence of neutrophil proteases such as elastase and cathepsin G in NET lysates degrades anticoagulants, including antithrombin III, heparin cofactor II, and TFPI, generating a pro-coagulant microenvironment and leading to activation of the coagulation cascade, facilitating platelet adhesion and aggregation, and providing structural scaffolds for thrombus formation (35). The process requires coordinated actions of peptidyl arginine deiminase 4 (PADI4), neutrophil elastase (ELANE), and myeloperoxidase (36) and is dependent on NADPH oxidase-derived reactive oxygen species and autophagy regulation.

Additionally, aPL monoclonal antibodies and β_2_GPI monoclonal antibodies in the serum of APS patients can enhance NETosis. Clinical studies demonstrated that elevated MPO-DNA complexes in thrombotic APS and SLE patients correlated with increased MPO and PADI4 mRNA expression (37). NETs have been confirmed to be associated with thrombosis in APS. Patients with primary APS have high levels of anti-NET antibodies, which hinder NET clearance and activate the complement cascade, thereby increasing thrombotic risk and aiding in risk stratification of APS (38).

### 2.3 Mechanism of complement activation

During tissue injury, exposed collagen on damaged blood vessel walls attracts platelet adhesion. Interactions between platelet surface receptors and ECM components induce platelet conformational changes, membrane phospholipid redistribution, and increased exposure of phosphatidylserine and chondroitin, thereby supporting complement activation (39, 40). Activated platelets degranulate, releasing coagulation factors such as factor V, FXI, FXIII, and complement components (41). Secondary hemostasis initiates through the TF pathway or the contact pathway; these pathways converge to produce activated FX (FXa), which then cleaves prothrombin into thrombin. Thrombin cleaves fibrinogen to form fibrin. Fibrin deposition exacerbates platelet thrombosis, with its regulation potentially influenced by the complement system function (42). Complement activation products (C3a, C5a, and C5b-9) induce ECs and monocytes to release pro-thrombotic factors and pro-inflammatory cytokines, resulting in overexpression of TF and molecules that facilitate platelet adhesion and coagulation (43). In individuals with positive aPL, aPL can independently activate ECs and platelets to participate in blood coagulation and induce monocyte TF expression and the generation of FXa upon activation of the TF pathway (44).

Beyond aPL, anti-NET and anti-FH antibodies also drive complement activation in APS. aPL initiates NET formation, elevating circulating NET levels in APS patients and facilitating complement activation (34, 45). Zuo et al. demonstrated elevated IgM anti-NET antibodies in primary APS patients, correlating with complement activation. This association likely stems from IgM antibody complement component interactions on NET surfaces (46, 47).

### 2.4 Lipid rafts in the immune signaling

Lipid rafts are cholesterol- and sphingolipid-enriched microdomains in the plasma membranes of endothelial cells, monocytes, and platelets. These highly ordered structures regulate cell polarity, vesicular trafficking, receptor aggregation, and signaling (48, 49). Upon interaction with aPL target antigens, lipid rafts initiate proinflammatory and procoagulant signaling cascades. This leads to excessive platelet activation, endothelial dysfunction, enhanced procoagulant states, and thrombophilia (50).

Hao Wei et al. demonstrated that activated platelet-derived EVs enriched in phosphatidylserine (PS) amplify thrombosis and inflammation through lipid raft-mediated mechanisms (51). Lipid rafts facilitate β_2_GPI antibody signaling by serving as platforms for Toll-like receptors TLR2, TLR4, and lipoprotein receptors LRP6 and LRP8/ApoER2. These interactions induce IRAK phosphorylation and NF-κB activation, promoting expression of TNF-α and TF (52, 53).

Gloria Riitano et al. demonstrated that disruption of lipid rafts with methyl-β-cyclodextrin (MβCD) or inhibition of LRP6 by Dickkopf-1 (DKK1) abolished IRAK phosphorylation and prevented formation of the β_2_GPI-LRP6-PAR-2 complex. This intervention significantly suppressed aPL-induced platelet activation, endothelial procoagulant responses, and proinflammatory activity, thereby inhibiting autoimmune thrombosis. Additionally, they discovered that β_2_GPI antibody activation of TLR8 induced eNOS phosphorylation and reduced intracellular NO levels (54, 55).

Collectively, this evidence establishes lipid rafts as essential platforms for initiating aPL/β_2_GPI complex-mediated signaling through TLR, LRP6, and LRP8 receptors.

### 2.5 Neutrophil granulocytes

In chronic inflammation, neutrophils exhibit significant phenotypic heterogeneity as terminally differentiated cells. The peripheral blood of SLE patients contains two distinct low-density granulocyte subsets. These are pro-inflammatory low-density granulocytes (LDG) and immunosuppressive myeloid-derived suppressor cells (PMN-MDSC). LDGs display reduced phagocytic activity but abnormally increased NET formation. By releasing inflammatory mediators such as interferon, IL-6, IL-8, and TNF-α, these cells exacerbate vascular complications in SLE and APS, acting synergistically with NET-mediated vascular endothelial injury. PMN-MDSCs exert immunosuppressive effects via regulatory lymphocyte pathways (56).

B lymphocyte dysfunction is central to the pathogenesis of primary APS. The low-affinity natural antibodies produced by CD5+ B cells may be involved in cross-reactivity to autoantigens. Plasmablasts, key precursors for antibody production, play an important role in aPL formation. Notably, β_2_GPI on the apoptotic cells’ surface can be recognized by specific B cells, leading to the activation of T-cell responses through antigen presentation. This process may involve MHC class II-mediated antigen processing mechanisms (57). The increased level of B-cell-activating factor (BAFF) is closely related to disease activity. Moreover, the up-regulation of BAFF mRNA expression in monocytes suggests that these cells may be the main source of circulating BAFF. BAFF promotes the survival and maturation of B cells by binding to BAFF-R, TACI, and BCMA receptors, thereby activating the nuclear factor-κB signaling pathway (58).

T-lymphocyte subset imbalance significantly contributes to APS pathogenesis. Peripheral blood proportions of helper T-cell subsets 1 and 17 (Th1 and Th17) are significantly increased, whereas regulatory T cell (Treg) numbers are reduced in thrombotic and obstetric APS patients. Moreover, oxidized β_2_GPI promotes the dendritic cells’ maturation, induces the differentiation of Th1 cells, and stimulates the release of pro-inflammatory cytokines (59). At the molecular level, β_2_GPI-reactive CD4+ T cells participate in thrombosis through IFN-γ-mediated monocyte activation and perforin and Fas ligand-dependent cytotoxicity. Additionally, specific β_2_GPI binding to NETs may exacerbate immune thrombotic events through epitope exposure mechanisms (60).

### 2.6 Microbial gut flora

The imbalance of exogenous pathogenic bacteria or endogenous strains in the body may induce infection and trigger aPL as a secondary event following the initial formation of autoantibodies (61). Humans harbor vast symbiotic microbial communities in the gut, skin, and oral cavity, forming a metabolic ecosystem that critically influences host immune regulation. When the intestinal barrier is compromised, the intestinal microbiome can disrupt the mucosal barrier and induce the production of mucosal-specific memory T cells. Microbiome-driven thrombosis in APS involves pro-inflammatory autoantibodies, bacterial translocation, antigen cross-reactivity, immune-modulating metabolites, and non-immune modifiers (62–64).

Compared with healthy individuals, it has been found that Bifidobacteria, Streptococci, and Clostridia related to intestinal inflammation and permeability are increased in patients with autoimmune diseases (63). Conversely, *Bilophila* in the *Coriobacteriaceae* decreases significantly whereas *Slackia* increases by 59%. The production of enteric cardiolipin can lead to the formation of pathogenic T-cell and autoantibody responses through cross-reactivity with autoantigens such as Ro 60, dsDNA, and β_2_GPI (65). Molecular simulations demonstrated that bacterial peptides homologous to β_2_GPI can induce pathogenic anti-β_2_GPI antibodies. The human intestinal bacterium *Roseburia* (*R. intestinalis*) possesses peptide sequences highly homologous to β_2_GPI, which induce pathogenic anti-β_2_GPI antibodies, which may stimulate lymphocytes and act as a chronic driver of β_2_GPI autoreactivity. However, gut microbiome effects on the APS may not depend solely on bacterial overgrowth, short-chain fatty acid production, or local inflammation. Vancomycin treatment in APS-prone (NZW × BXSB) F1 mice reduces anti-β_2_GPI antibody production, accompanied by lower Th17 and T follicular helper cell frequencies in secondary lymphoid tissues and reduced autoantibody levels (64).

SLE patients demonstrate reduced gut microbiota richness. Both the *Firmicutes/Bacteroidetes* ratio and fecal *Lactobacillus* abundance are significantly decreased in SLE cohorts (n=20), with reductions correlating with disease activity, particularly lupus nephritis (66, 67). In addition, *Microbacterium* and *Pseudobutyrivibrio* are reduced in SLE patients. Microbiota dysbiosis is related to local inflammatory responses, high circulating levels of anti-dsDNA and histone antibodies, and systemic immune activation, which promotes lymphocyte activity and Th17 cell differentiation (64).

*Lactobacillus plantarum* improves renal function in murine lupus nephritis models. Intestinal lactic acid bacteria colonization restores mucosal barrier function, reduces IL-6 and IL-18, and mitigates kidney damage by decreasing immune complex deposition and IFN-γ levels (68). Supplementation of *Lactobacillus* casei (Actimel) or yogurt in APS animal models effectively inhibited IL-10 production (P < 0.05) and enhanced IFN-γ secretion. Metagenomic analysis of 124 SLE patients compared with healthy populations showed significant associations between *Escherichia coli*, purine nucleotide metabolism, and peptidomature metabolic pathways with the SLE disease activity index (SLEDAI), as well as between multiple *Escherichia coli* epitopes and disease activity or renal involvement phenotypes (69).

### 2.7 Genetic features

The pathogenesis of APS involves both genetic susceptibility and environmental triggers. The Val247 variant is associated with anti-β_2_GPI antibody production in primary APS patients, and STAT4 SNP T allele frequencies are significantly higher in Japanese SLE and APS patients than in healthy controls (70, 71). Among 276 upregulated genes in APS, enriched Gene Ontology (GO) terms included innate immune response, leukocyte activation, lymphocyte activation, cytokine production, T-cell activation, complement activation, and NET gene expression (72). Current evidence indicates that APS-associated genetic variants cluster primarily within the human leukocyte antigen (HLA) system and involve specific single-nucleotide polymorphisms (SNPs). HLA-DR and HLA-DQ alleles contribute to APS susceptibility through genetic and epigenetic mechanisms (73). The syndrome appears to involve two dominant complementary alleles localized to separate chromosomes (74). The expression of HLA alleles such as HLA-DR4, -DR7, -DRw53, and -DQB1*0302 is associated with aPL and is observed in both primary APS and APS secondary to SLE (75).

APS and SLE are frequently comorbid, with 40% of SLE patients testing positive for aPL. In contrast, primary APS often presents with the presence of circulating antinuclear antibodies or anti-dsDNA/chromatin antibodies. 30% to 50% of patients with SLE have been reported to test positive for at least one aPL test (76). Despite shared lupus susceptibility genes, primary APS patients rarely progress to full SLE even after a decade of follow-up, underscoring their distinct pathogenesis, likely due to differing immunological mechanisms (77, 78). Early observations by Harvey and Shulman suggested a genetic basis for aPLs, noting familial clustering of syphilis false-positive tests among SLE and primary APS patients with multiple affected first-degree relatives. These findings implied that genetic susceptibility promotes aPL expression and that extended kinship correlates with APS manifestations, supporting APS as a familial disorder (71).

### 2.8 Pathophysiology of pregnancy complications

Vascular thrombosis was initially regarded as the primary cause of pregnancy complications in APS. Current evidence from animal models demonstrates that aPL-mediated placental dysfunction and complement-driven inflammatory processes represent the predominant pathogenic mechanisms underlying adverse obstetric outcomes (79). During placenta formation, extensive trophoblast remodeling exposes anionic phospholipids on cell surfaces, facilitating aPL and β_2_GPI complex formation (80). Further changes in trophoblastic cell function include reduced cell proliferation and migration, increased production of pro-inflammatory cytokines, and decreased secretion of human chorionic gonadotropin and pro-angiogenic factors such as vascular endothelial growth factor (VEGF) (81, 82). In addition, aPLs suppress endometrial angiogenesis both *in vitro* and *in vivo* by inhibiting endothelial cell differentiation and VEGF expression during neovascularization (83). Moreover, Marder et al. demonstrated that anti-β_2_GPI antibodies bind to the surface of neutrophils and increase the release of NETs. NETs serve antimicrobial functions, and NETosis promotes sterile inflammation. In pregnant women with systemic lupus erythematosus, APS, and preeclampsia, a higher number of NETs in the placental villous space are associated with significant inflammation and vascular modification (84). Placental trophoblast-derived extracellular vesicles (EVs) contribute to APS pathogenesis by modulating decidual and maternal endothelial cell function (85). Notably, Wu et al. demonstrated that aPL-stimulated endothelial cells release EVs capable of activating TLR7-mediated inflammation via IL-1β and single-stranded RNA molecules (86).

Platelet activation: Antiphospholipid antibodies (aPLs) bind β_2_-glycoprotein I (β_2_GPI) complexes, engaging platelet surface receptors (GPIb-IX-V, GPIIb/IIIa) via lipid rafts and LDL receptor family members (LRP6, LRP8). This triggers platelet activation, promoting aggregation and impairing fibrinolysis.

Neutrophil-mediated inflammation and thrombosis: aPL/β_2_GPI complexes activate TLR4 and C5aR, inducing NF-κB through the MyD88 pathway. This upregulates proinflammatory cytokines (TNF-α, IL-6, IL-8) and enhances ROS and tissue factor (TF) production. NETosis (neutrophil extracellular trap formation) further amplifies inflammation and coagulation via PI3K/AKT/mTOR signaling, with adhesion molecules (E-selectin, VCAM-1, ICAM-1) facilitating endothelial interaction.

Monocyte procoagulant activity: aPL/β_2_GPI binding to TLR8, TLR6, and LDL receptors (LRP6/LRP8) activates NF-κB, driving TNF-α, IL-1β, IL-6, and TF expression, whereas ROS generation exacerbates endothelial dysfunction.

Lymphocyte dysregulation: aPLs stimulate lymphocytes to secrete IFN-γ and BAFF, promoting B-cell activation and autoantibody production, which sustains immune dysregulation.

Endothelial dysfunction and coagulation imbalance: activating complement (C5a, C3a, MAC) and impairing thrombomodulin (TM) and protein C pathways. Reducing NO and PGI2, while upregulating TF and proinflammatory cytokines (TNF-α, IL-6); dysregulating fibrinolysis via plasminogen, plasmin, PAI-1, and FDP, leading to a prothrombotic state.

## 3 Clinical manifestations

### 3.1 Epidemiology

The global prevalence of APS is approximately 2%, with recent studies demonstrating consistent epidemiological trends. A 2019 study showed that the annual incidence of APS was 2.1%; the incidence ratio between women and men is approximately 3.5:1. Incidence peaks after age 55, particularly beyond 75 years (87). A population-based cohort study from Olmsted County, Minnesota, estimated that the annual incidence of APS was 2.1 patients per 100,000 population, with a prevalence of 50 cases per 100,000 population (88). In a 2019 epidemiological study of individuals over the age of 18, the incidence of APS was estimated at 2.1 cases per 100,000 people, with an estimated prevalence of 50 cases per 100,000 (87).

### 3.2 Classification criteria

**Figure 2:**
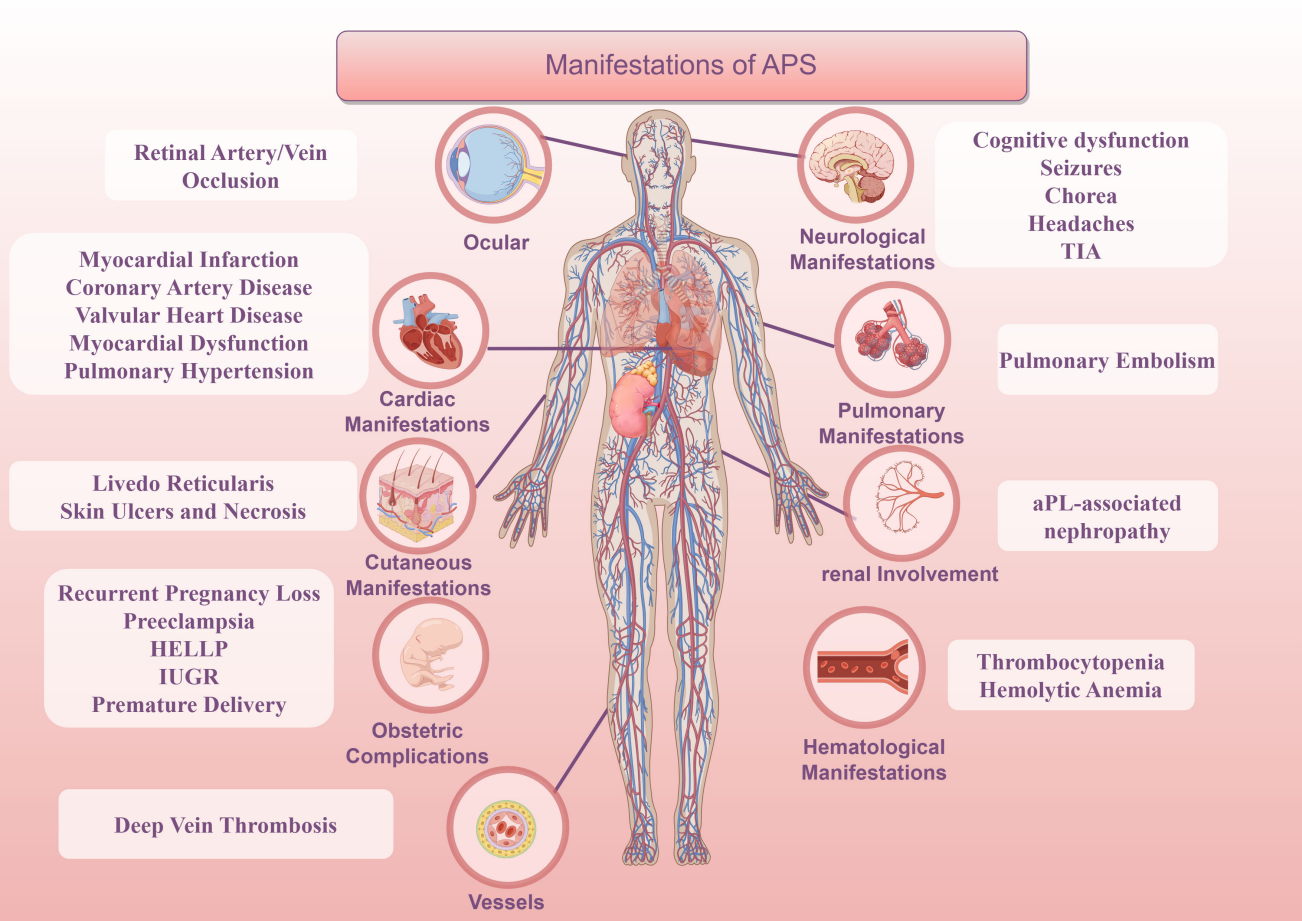
Manifestation of APS.

The preliminary classification criteria were first established at an expert seminar held in Sapporo, Japan, in 1999. At a 2006 meeting in Sydney, Australia, experts proposed several changes to the previous classification criteria, such as adding tests for aCL and anti-β_2_GPI antibodies, extending the time interval between serological tests, and providing clearer definitions of clinical presentations and laboratory titer thresholds (89, 90). Specific features associated with APS were highlighted, including heart valve involvement, reticular cyanosis, thrombocytopenia, APS nephropathy, and non-thrombotic central nervous system manifestations (**Figure 2**).

The 2023 ACR/EULAR APS classification criteria include an entry criterion of at least one positive aPL test within 3 years of identifying the clinical criteria associated with aPL. These criteria are divided into six clinical domains—macrovascular venous thromboembolism, macrovascular arterial thrombosis, microvascular thrombosis, obstetrics, heart valve involvement, and hematology—and two laboratory domains: lupus anticoagulant functional coagulation assay and solid-phase ELISA tests for detecting IgG/IgM anti-cardiolipin and/or IgG/IgM anti-beta-2 glycoprotein I antibodies. Patients who accumulate at least three points in total from the clinical and laboratory criteria are classified as having APS (90). Compared with the Sapporo classification criteria revised in 2006, the new APS criteria demonstrate increased specificity from 86% to 99% and sensitivity from 84% to 99% (91).

Besides SLE, APS can occur secondary to malignant tumors, infections, and blood disorders such as autoimmune diseases. Antiphospholipid antibodies can be detected in approximately half of APS patients. The coexistence of APS and SLE is associated with increased mortality and poorer clinical outcomes. Therefore, early diagnosis and risk assessment.

### 3.3 Common manifestations

APS is characterized by venous thrombosis and arterial thrombosis. APS can be divided into different phenotypes according to clinical manifestations, with thrombotic APS being more common. The main manifestation of venous thrombosis is deep vein thrombosis (DVT) of the lower extremity, which may lead to pulmonary embolism (PE). Arterial thromboses can cause ischemic stroke, transient ischemic attacks (TIAs), myocardial infarction, and thromboses in atypical sites such as renal veins, mesenteric arteries, and upper limbs. Vascular APS is a type that emphasizes thrombotic events as the core pathological mechanism; thrombosis leads to acute or chronic vascular obstruction, resulting in tissue damage and organ dysfunction.

Hematologic abnormalities include prolonged activated partial thromboplastin time, thrombocytopenia, and thrombotic microangiopathy. While leukopenia, lymphocytopenia, and autoimmune hemolytic anemia (AIHA) overlap with SLE, catastrophic APS (CAPS) and thrombotic thrombocytopenic purpura (TTP) distinguish APS severity (92, 93). Thrombocytopenia occurs in 15% of patients, typically mild but posing surgical or traumatic bleeding risks in severe cases. Neurological sequelae include stroke, TIAs, cognitive dysfunction, migraines, and rare seizures (94). In the latest ACR/EULAR classification criteria, thrombocytopenia was included in the clinical criteria, possibly due to increased depletion and destruction of peripheral blood platelets following aPL activation. Additionally, 29% of patients with SLE-associated APS had a significant history of thrombocytopenia (90). Systemic inflammatory manifestations in COVID-19 patients contribute to APS induction, and the cumulative effect of aPL further promotes this process (95).

Lupus antiphospholipid syndrome, also known as secondary APS, may occur in patients with systemic lupus erythematosus. It is characterized by one or more positive aPL, including LA, aCL, and/or anti-β_2_GPI antibodies, detected over a period of at least 12 weeks. There is one or more typical clinical event associated with the syndrome. CAPS is an extreme variant of APS, characterized by a sudden onset of generalized thrombosis affecting multiple-organ systems, including vital organs such as the lungs, kidneys, brain, and heart.

In contrast to the above criteria, some patients present with clinical manifestations such as widespread thrombosis, recurrent arteriovenous thrombosis events, recurrent miscarriage, or unexplained thrombocytopenia, accompanied by aPL titers that are only temporarily positive or persistently negative. This type of APS, characterized by “non-criteria” clinical manifestations, is defined as SN-APS after excluding other causes of thrombosis, such as hereditary thrombophilic predisposition, including prothrombin factor V and factor II mutations, coagulation disorders, active cancer, trauma, major surgery, and prolonged bed rest (30).

### 3.4 Complications

#### 3.4.1 Pulmonary manifestations

Since the early 1990s, autoimmunity has been hypothesized to contribute to pulmonary hypertension (PH) pathogenesis. Pathological features observed in PH include plexiform lesions in small pulmonary arteries and fibrinoid vasculitis affecting all layers of medium-sized arteries. Microthrombosis has been documented in pulmonary biopsies of SLE patients with aPL or primary APS, where pulmonary arterioles exhibit thromboembolic “mesh lesions”. Although plexiform lesions characterize severe PH-associated pulmonary artery disease, they are non-specific and may arise from recurrent pulmonary embolism (PE), pulmonary capillaritis, or idiopathic pulmonary arterial hypertension (IPAH). Notably, these lesions occur in aPL-positive patients, suggesting a link between aPL positivity and endothelial remodeling. A significant proportion of patients with idiopathic pulmonary arterial hypertension (PAH) test positive for aPL (10% to 44%) (96).

In a systematic review and meta-analysis of 31 studies that included 4,480 patients with SLE and 410 cases of PH, it was first demonstrated that SLE patients with aPL had a twofold higher risk of PH compared with patients without aPL. Additionally, aPL carriers have a doubled risk of developing aPL-associated PAH, with specific aPL profiles (LA or IgG aCL) conferring higher PH risk (97).

#### 3.4.2 Cardiovascular manifestations

Cardiac manifestations in primary APS are well-established, with valvular lesions confirmed as a core feature. Coronary artery disease is also prevalent in APS; the European Phospholipid Project’s study of 1,000 APS patients reported acute myocardial infarction rates of 4%–7%, often comorbid with SLE. Approximately 20% of primary APS patients exhibit cardiac involvement, with CAD having the highest prevalence (10.2%), followed by valvular disease (8.9%). Subanalysis revealed significant associations between LA positivity and myocardial infarction (p=0.031) and a potential link between aCL-IgG and valvular pathology (p=0.041) (98).

#### 3.4.3 Renal complications

The kidney serves as a primary target organ in APS, with vascular damage spanning large, medium, and small vessels, including capillaries in primary, secondary, and CAPS. Renal presentations vary from asymptomatic mild proteinuria to hypertension, alongside nephrotic-range proteinuria, active urinary sediment, or renal impairment. Pathological alterations involve renal infarction, arterial thrombosis, and venous thrombosis occurring in both primary and secondary disease, which may exacerbate preexisting proteinuria or trigger new-onset nephrotic syndrome, hypertension, or renal failure (99–101).

Antiphospholipid syndrome nephropathy (APSN) involves thrombotic microangiopathy affecting glomerular, interstitial, and peritubular vessels. Histopathological features encompass acute or chronic lesions such as fibrosis, tubular thyroidization, focal cortical atrophy, and glomerulosclerosis. APS may also cause isolated glomerulonephritis, most commonly membranous nephropathy. Critically, persistent aPL positivity adversely impacts end-stage renal disease management, correlating with kidney transplant failure. APS with lupus nephritis further worsens transplant outcomes versus lupus nephritis alone, increasing risks of allograft loss, delayed graft function, and acute rejection (99, 100).

#### 3.4.4 Cutaneous manifestations

aPL-positive SLE patients exhibit higher risks of reticulate purpura compared with antibody-negative individuals. Although reticulate purpura frequently occurs in SLE patients with small vessel disease, its presentation as an initial lesion remains uncommon; nevertheless, such cases necessitate early diagnosis and aggressive intervention. Notably, in CAPS, skin changes accompanying multiorgan failure provide critical diagnostic value (102).

#### 3.4.5 Neurologic manifestations

SLE and APS constitute serious causes of neurological disorders, with aPLs recognized as risk factors for ischemic stroke and recurrent thrombosis. Evidence indicates that these antibodies activate cerebral endothelium, enhancing procoagulant activity and neuronal injury through endothelial adhesion molecule upregulation and proinflammatory cytokine release. A systematic review of 43 studies encompassing 5,217 patients and controls demonstrated aPL involvement in 17.4% of cerebrovascular events, 17.2% of strokes, and 11.7% of transient ischemic attacks, conferring a fivefold increased stroke or transient ischemic attack risk (103). Cerebral venous sinus thrombosis and stroke represent rare but established APS manifestations, with cerebral venous sinus thrombosis first documented as an initial presentation in 2009. Headache, visual impairment, and cognitive dysfunction constitute primary symptoms in APS-associated cerebral venous sinus thrombosis, occurring at frequencies of 85%, 40%, and 25%, respectively (104).

#### 3.4.6 Ocular manifestations

Over 25% of asymptomatic APS and SLE patients display retinal abnormalities. Drusen-like deposits represent the most prevalent finding, showing comparable frequency in both conditions, whereas acute paracentral acute middle maculopathy lesions occur specifically in these patients. The association of paracentral acute middle maculopathy with triple-positive antiphospholipid antibody profiles and high adjusted Global Antiphospholipid Syndrome Score values suggests these factors may drive macular degeneration pathogenesis (105, 106).

#### 3.4.7 pAPS

Pediatric antiphospholipid syndrome (pAPS) affects individuals under 18 years of age. While its clinical manifestations resemble those in other age groups, pAPS may exert additional impacts on growth and development due to its occurrence during childhood. Although more prevalent in adults, APS occasionally presents neonatally or during childhood. Pediatric studies reveal distinct disease patterns characterized by non-criteria manifestations such as Evans syndrome, Raynaud’s phenomenon, migraines, and chorea. Thromboembolic events predominantly involve deep vein thrombosis, stroke, and pulmonary embolism in children. Notably, the female predominance observed in adult-onset APS is less pronounced in pediatric cases, with more balanced gender distribution. Children exhibit higher recurrent thromboembolism risks than adults but lower prevalence of livedo reticularis-related symptoms (107).

Children initially diagnosed with primary APS may develop SLE over time. Studies report aPL prevalence in pediatric APS patients as 44% for aCL antibodies, 40% for anti-β_2_GPI antibodies, and 22% for LA. Consequently, secondary APS screening is recommended for all adolescents with SLE (108).

#### 3.4.8 OAPS

Obstetric antiphospholipid syndrome (OAPS) is defined by either unexplained death of one or more morphologically normal fetuses at or beyond 10 gestational weeks, or premature birth before 34 weeks due to eclampsia, severe preeclampsia, or placental insufficiency, or alternatively, three or more consecutive spontaneous abortions prior to 10 gestational weeks after rigorous exclusion of parental chromosomal abnormalities, maternal anatomical defects, and hormonal etiologies (109).

Clinical evidence regarding the gender-based differences in APS outcomes demonstrates that primary APS exhibits a higher prevalence in male patients compared with females (p<0.001). Male patients show significantly greater incidence of peripheral artery thrombosis (p=0.049) and myocardial infarction (p=0.031) (110). In primary APS, male patients typically present with a more limited clinical spectrum dominated by thrombosis and thrombocytopenia, yet their prognosis remains poorer with greater therapeutic challenges. Importantly, male gender itself constitutes an independent risk factor for adverse outcomes (111). Gender further influences thrombosis patterns in primary APS, with male patients characteristically developing arterial thrombotic events at a later disease stage, whereas no significant serological differences in the frequency or titer of different antiphospholipid antibodies exist between genders (112).

Primary obstetrical APS refers to specific pregnancy complications experienced by a woman due to the presence of aPLs in the absence of other known autoimmune diseases. Women who are positive for aPL have a risk for pregnancy complications (113). aPLs primarily induce placental thrombosis and infarction, leading to recurrent complications including early miscarriage, fetal loss, early-onset preeclampsia, IUGR, placental abruption, and preterm delivery. Patients with aPLs or systemic lupus erythematosus experience higher pregnancy complication rates, with HELLP syndrome occurring earlier and predominantly in the second trimester compared with the general obstetric population (109, 114).

## 4 Therapy and prevention

Prophylactic anticoagulant therapy represents the cornerstone of thrombotic APS management in patients at high risk of thrombotic events. Anticoagulant therapy in APS includes direct oral anticoagulants (DOACs) and antiplatelet drugs. Parenteral anticoagulant options include low molecular weight heparin (LMWH), unfractionated heparin (UFH), and fondaparinux. Symptomatic treatments include hydroxychloroquine (HCQ), statins, and vitamin D. Targeted therapies include rituximab, belimumab, anti-TNF agents, complement inhibitors, and β_2_GPI peptide-based drugs (115–117). A brief overview is presented in **Table 1**.

**Table 1:** Treatment strategies for aPL-positive patients.

Classification	Medications	Mechanism	Recommended dose	Suitable for the crowd	Announcements
Antiplatelet agent	LDA (207)	Cyclooxygenase inhibitor	75–100 mg/day	All APS patients (especially obstetric APS); primary prevention for all APS patients in asymptomatic APL-positive individuals	Use throughout pregnancy; discontinue 7–10 days before delivery
	Abciximab	Glycoprotein IIb/IIIa receptor inhibitor	Insufficient clinical evidence		
Anticoagulant	LMWH (208–210)	Indirect thrombin inhibitor	Preventive dosage: 40–60 mg/day Therapeutic dosage: 1 mg/kg every 12 h (acute stage of thrombosis)	Obstetric APS; acute thrombosis; postpartum anticoagulation (6–12 weeks)	Monitor anti-Xa activity in pregnancy and renal impairment
	Warfarin (207, 211)	Vitamin K antagonist	Initial: 2.5–5 mg/day Maintenance: INR 2.0-3.0 (venous thromboembolism) INR 3.0-4.0 (recurrent arterial thrombosis)	Long-term anticoagulation for thrombotic APS in non-gestational periods Postpartum anticoagulation can replace LMWH	Teratogenic; contraindicated in pregnancy
	DOACs (rivaroxaban, dabigatran) (212)		Insufficient clinical evidence		
	Fondaparinux (213, 214)	Selective factor Xa inhibitor	Insufficient clinical evidence	Basic treatment for all APS patients; Prevents the recurrence of thrombosis	Limited pregnancy safety data
Immunomodulator	Hydroxychloroquine (215, 216)		200–400 mg/day	Basic treatment for all APS patients; thrombosis recurrence prevention	Relatively pregnancy-safe; annual retinal screening required
	Glucocorticoid (217)		Prednisone: 1–2 mg/kg/day	Refractory OAPS CAPS; combined with thrombocytopenia/hemolytic anemia;	Limit to ≤6 weeks; long-term use increases diabetes and osteoporosis risk
	Intravenous immunoglobulin (217–219)		400 mg/(kg·day) × 3–5 days	Refractory OAPS CAPS; severe thrombocytopenia	Second-line therapy
	Plasma exchange (217, 220)		Insufficient clinical evidence	First or second-line for catastrophic APS; acute thrombotic microangiopathy in aPL-nephropathy	
	Rituximab (221, 222)	Type I anti-CD20 monoclonal antibody	375 mg/m²/week ×4 weeks or 1000 mg biweekly ×2 doses	Standard treatment is ineffective CAPS; refractory thrombosis/thrombocytopenia	Discontinue use during the first 6 months of pregnancy; infection risk
	Belimumab (223)	BAFF/BLyS inhibitor	Insufficient clinical evidence		
	Sirolimus (224)	mTOR Inhibitors	1–2 mg/day (target concentration ≤15 ng/mL)	Thrombocytopenia in primary APS	
Complement inhibitor	Eculizumab (Hussain, 2022 #12) (225)	Complement 5 inhibitor	600 mg/week ×4→900 mg/2 weeks	Refractory CAPS; thrombotic microangiopathy	Require meningococcal vaccination; infection monitoring
Cholesterol-binding agents	Statins (178)	hmg-coa reductase inhibitors	Insufficient clinical evidence		

### 4.1 Thromboprophylaxis

#### 4.1.1 Primary prevention

Primary prevention for the initial thrombosis and secondary prevention for recurrence of thrombosis in patients with a history of thrombosis should be followed according to risk stratification. Prior to anticoagulant therapy, clinicians should assess aPL profile and cardiovascular risk factors using validated scoring systems to weigh treatment benefits against risks (118).

In asymptomatic aPL-positive SLE patients without prior thrombosis or obstetric complications, low-dose aspirin (LDA, 75 mg–100 mg daily) is recommended for prophylactic therapy. The annual risk of thrombosis in aPL-positive patients without previous thrombosis ranges from 0% to 3.8%. According to thrombotic risk stratification, regular monitoring, elimination of non-aPL thrombotic risk factors, and active management of both clinical and subclinical systemic autoimmune disease activity are required. For patients with low-risk aPL profiles, LDA may be considered for prophylactic therapy; however, its specific efficacy in preventing thrombotic events has not been conclusively established (119).

#### 4.1.2 Secondary prevention

Only patients with persistent medium/high titer positivity or multiple positive aPL should be considered high-risk and receive antiplatelet primary prophylaxis, especially if additional risk factors for thrombosis are present (120). For patients with unprovoked first venous thrombosis, long-term anticoagulation therapy should be continued (121). In patients with definitive APS and first-time venous thrombosis, vitamin K antagonist (VKA) therapy with a target international normalized ratio (INR) of 2–3 is recommended.

DOACs, which include oral direct thrombin inhibitors (dabigatran) or direct Factor Xa inhibitors (rivaroxaban, edoxaban, or apixaban), may be considered (5/D) for patients who are unable to achieve their target INR despite good VKA adherence or who have contraindications to VKA. Non-vitamin K antagonist oral anticoagulants have been identified as therapeutic alternatives to VKA for VTE treatment and secondary prevention in patients without APS. If VKA contraindications are present or the INR is poorly controlled, DOACs may be an option for patients with APS and first-time VTE, who are typically treated with standard-intensity VKA. DOACs offer advantages such as simplified oral administration, no food interactions, and no requirement for INR monitoring. However, several meta-analyses have shown a significantly increased risk of arterial thrombosis (AT) with DOAC treatment compared with VKA in the prevention of thrombosis (122), and this disadvantage is also significant in patients with a history of venous thrombosis. The European Medicines Agency (123), following a risk assessment triggered by Rivaroxaban for thrombotic APS randomized controlled trials, has stated that DOACs are not recommended for patients with thrombotic APS, especially those with triple aPL positivity. A multicenter, prospective, randomized controlled trial also showed that apixaban had a higher incidence of stroke than warfarin and was not a routine alternative to warfarin (124). A total of 624 patients were included in five randomized controlled trials that met the inclusion criteria. The primary outcome measure was a new thrombotic event during the VKA-controlled treatment period, defined as the composite endpoint of any VT or AT. Among 305 and 319 APS patients treated with DOACs and VKA, respectively, there was a significantly increased risk of AT with DOAC treatment compared with VKA (OR 5.5, 95% CI 2.5–12.1; p < 0.0001), but no difference in VT risk (p = 0.87) (125). Based on current evidence, we do not recommend the use of DOACs in patients with definitive APS and arterial events because of the higher risk of recurrent thrombosis (122, 126).

In patients who still develop APS and recurrent venous thrombosis after VKA treatment, LDA can be increased, or LMWH dosage can be adjusted if the INR is within the therapeutic range.

### 4.2 Immunomodulator

#### 4.2.1 Hydroxychloroquine

Hydroxychloroquine (HCQ) was developed in 1995 by modifying the hydroxyl group of chloroquine, originally extracted from Cinchona bark for malaria treatment. Beyond malaria, HCQ is widely used for conditions affecting metabolic, cardiovascular, hematologic, and autoimmune systems, as well as bacterial and viral infections, including rheumatoid arthritis, SLE, dermatomyositis, and Sjögren’s syndrome. As a TLR7 and TLR9 antagonist, HCQ has anti-inflammatory, anti-aggregation, and immunomodulatory properties. Its mechanisms may include stabilizing lysosomal membranes by inhibiting phospholipase activity, blocking aPL binding to cells, raising extracellular and intracellular pH, and inhibiting complement-dependent antigen-antibody responses (127, 128). Placental toxicology studies indicate no adverse effects on placental tissue and suggest potential anti-inflammatory protection. Experimental models demonstrate that HCQ can restore the biological function of aPL-damaged trophoblastic cells and protect against placental and fetal neurodevelopmental abnormalities (129). HCQ reduces aPL-induced platelet aggregation and coagulation, inhibits anti-β_2_GPI antibody formation, and lowers thrombosis risk. Studies suggest that HCQ may reduce aPL titers in SLE patients and APS animal models, effectively preventing thrombosis (130).

Current guidelines recommend adding HCQ to aspirin in aPL-positive SLE patients. For OAPS, combining HCQ with aspirin improves outcomes, with additional HCQ reducing pregnancy loss and increasing live birth rates (131, 132). A retrospective single-center cohort study of women with refractory primary OAPS found that adding HCQ (400 mg) to enoxaparin (60 mg) and LDA significantly increased live birth rates and decreased complication rates (RR: 1.55, 95% CI 1.19–2.1; p < 0.001) (133). Additionally, HCQ may reduce the risk of lupus activity flare during pregnancy and preeclampsia incidence in SLE patients (134).

The HIBISCUS trial—a multicenter, prospective, randomized, double-blind, placebo-controlled study evaluating HCQ for secondary prevention of obstetric and thrombotic events in primary APS—is underway. HCQ currently holds orphan drug designation for APS from the European Medicines Agency (135). While HCQ shows promise in refractory OAPS, its mechanisms remain incompletely understood, requiring further real-world efficacy data.

#### 4.2.2 B cell inhibitors

##### 4.2.2.1 Anti-CD20 monoclonal antibody

Primary APS patients exhibit altered B-cell subsets characterized by increased plasma cells and elevated BAFF levels. Human and murine studies indicate that B-cell-targeted therapy confers clinical and serological benefits, including reduced aPL titers post-treatment (136). Rituximab, which targets the CD20 antigen on B cells, is clinically used for B-cell non-Hodgkin lymphoma and has been increasingly applied to autoimmune conditions such as SLE, autoimmune hemolytic anemia (AIHA), and vasculitis. Previous studies demonstrate that combining rituximab with anticoagulation, glucocorticoids, and plasma exchange achieves favorable outcomes in refractory CAPS thrombosis and recurrent thrombotic events (137–139). Notably, rituximab therapy induces complete remission of severe thrombocytopenia (defined as >150,000 platelets/mm³) in 83.3% of APS patients with this complication (140).

Current guidelines reserve rituximab for refractory CAPS cases due to insufficient evidence supporting its first-line use. For SLE-specific manifestations, alternative second-line agents include baricitinib, bortezomib, eculizumab, anakinra, and tocilizumab (141).

##### 4.2.2.2 BAFF/BLyS inhibitors

Belimumab inhibits BAFF by blocking its binding to B-cell receptors. Large phase III trials demonstrated clinical efficacy against musculoskeletal, mucocutaneous, hematological, and systemic manifestations of SLE, leading to its FDA approval for SLE treatment (142). In NZW×BXSB F1 mice, belimumab prevented myocardial infarction and nephritis and delayed thrombocytopenia, although it failed to inhibit anti-cardiolipin antibody formation (143). This agent is also applicable in severe SLE and APS cases (141). Belimumab reduces aPL titers in primary triple-positive APS patients (144), and an open-label, prospective phase II trial evaluating its use for refractory or non-standard APS manifestations is currently ongoing (145). Multiple real-world case reports confirm that belimumab treatment for refractory aPL-associated immune thrombocytopenia increases platelet counts and modulates aPL levels (146, 147).

#### 4.2.3 Complement inhibitors

Eculizumab, a recombinant humanized IgG2/IgG4 monoclonal antibody, binds complement protein C5 to inhibit its cleavage into C5a and C5b, thereby preventing terminal complement complex C5b-9 formation (148). Case reports demonstrate its efficacy in controlling complement overactivation in refractory APS and CAPS (149, 150). Based on terminal complement inhibition principles, a U.S. study found preemptive eculizumab administration before kidney transplantation prevents vascular injury and thrombosis, effectively averting post-transplant APS (151). Currently, eculizumab is successfully used to prevent and treat APS-related thrombotic microangiopathy (TMA) in renal transplant recipients.

The international CAPS Registry analysis documented 584 eculizumab-treated patients, with the most common regimen being 900 mg weekly for 4 weeks followed by 1,200 mg biweekly (152). Refractory secondary TMA case reports show significant improvements in platelet counts, lactate dehydrogenase (122), and estimated glomerular filtration rate (eGFR) after 4 weeks of eculizumab therapy. Positive outcomes were also observed in LN patients with TMA, including a documented case where a 30-week pregnant APS patient avoided thrombosis and organ damage following treatment (153, 154). Consequently, eculizumab represents a first-line option for SLE, APS, or LN-associated TMA (155).

#### 4.2.4 mTOR inhibitors

Sirolimus, also known as rapamycin, was first isolated from *Streptomyces hygroscopicus* in 1972 and regulates cell growth, proliferation, and survival by inhibiting the mammalian target of rapamycin (mTOR) pathway. This pathway is associated with intimal hyperplasia development and peripheral vascular smooth muscle cell proliferation. Sirolimus additionally inhibits antigen-induced T-cell proliferation, increases circulating regulatory T cells, and blocks T-cell activation (156). mTOR pathway activation contributes to SLE pathogenesis, where mTORC1 activation precedes clinical SLE and APS, serving as an early disease marker (157).

Single-subject, open-label phase I/II trials and real-world studies confirm oral sirolimus’s safety and efficacy in SLE, demonstrating improved serological parameters and glucocorticoid tapering (158–160). Poznyak et al. reported that sirolimus treatment restored T-lymphocyte subpopulation balance, improving clinical manifestations, serological abnormalities, and disease activity in SLE patients. This multitarget therapy increases Treg cell numbers, corrects Treg/Th17 imbalance, modulates anti-inflammatory/pro-inflammatory cytokines, restores mitochondrial homeostasis, reduces oxidative stress, regulates immune responses, decreases autoantibody titers, and ameliorates organ damage (161).

A Chinese case report established sirolimus monotherapy efficacy in refractory lupus nephritis (LN) with APS characterized by mTOR pathway activation. A phase I/II trial showed that 12-month sirolimus treatment expanded Treg and CD8+ memory T cells while inhibiting interleukin-4 and interleukin-17 production. Retrospective analysis of post-renal transplant patients revealed sirolimus inhibited renal vascular AKT/mTOR signaling in APS nephropathy, significantly improving renal function and vascular pathology while confirming endothelial mTORC activation molecularly, with good tolerability (162). Data further indicate that sirolimus significantly alleviates SLE musculoskeletal symptoms, including arthritis and tenosynovitis, while also improving cutaneous and mucosal manifestations, primarily through reducing rash frequency and severity (158).

### 4.3 Management of special population

#### 4.3.1 OAPS treatment

For women with high-risk aPL profiles but no history of thrombosis or pregnancy complications, prophylactic antithrombotic therapy with LDA, 75–100 mg daily, should be considered during pregnancy, particularly given a history of obstetric APS.

Standard obstetric APS is diagnosed in women with ≥3 recurrent spontaneous abortions before 10 weeks of gestation or fetal loss at or beyond 10 weeks, eclampsia, severe preeclampsia, or placental insufficiency. “Non-standard” obstetric APS includes cases like two recurrent spontaneous abortions before 10 weeks of gestation or delivery at ≥34 weeks due to severe preeclampsia or eclampsia. For both presentations, combination therapy with LDA and prophylactic-dose heparin during pregnancy is recommended, with prophylactic-dose heparin continued for 6 weeks postpartum to prevent maternal thrombosis. Randomized controlled studies demonstrate that LDA combined with LMWH improves live birth rates and reduces APS-related complications in pregnant women with recurrent miscarriage (163). Although standard treatment reduces adverse obstetric outcomes, some OAPS patients remain at risk for preeclampsia, thrombosis, preterm birth, and childhood neurodevelopmental impairments. A retrospective study of 102 OAPS patients identified triple aPL positivity (OR=24.70, 95% CI 4.27-142.92, p<0.001) and prior early severe preeclampsia (OR=7.11, 95% CI 1.13-44.64, p=0.036) as significant risk factors for early severe preeclampsia (164). Control of comorbidities such as thrombosis and hypertension is also crucial for preventing adverse pregnancy outcomes.

Beyond traditional therapy, options include increasing heparin to therapeutic intensity, adding HCQ or low-dose prednisolone in early pregnancy, or administering intravenous immunoglobulin (IVIg) (165–167). Agents such as coenzyme Q10, dipyridamole, and histone deacetylase inhibitors may inhibit tissue factor expression or modulate angiogenic balance, but supporting clinical evidence remains insufficient, requiring further study. OAPS presents with non-specific, easily overlooked symptoms but can have severe onsets. Plasma exchange for pregnancy-induced CAPS targets aPLs, pro-inflammatory factors, and pro-thrombotic mediators. The typical protocol involves 2-3 L of plasma exchange over 3–5 days. Early intervention before microvascular thrombosis and organ failure is critical for pregnant patients (168).

#### 4.3.2 CAPS treatment

CAPS is characterized by a “thrombotic storm,” presenting as vascular occlusion and an “inflammatory storm” affecting multiple-organ systems within a short period. Histopathology shows small vessel occlusion in at least one organ or tissue, with serological manifestations of elevated aPL titers (169). Anticoagulation, plasma exchange, and high-dose corticosteroid triple therapy are standard treatments for CAPS. When CAPS risk is identified, timely prevention or intervention should target triggers including infection, surgery, trauma, anticoagulant withdrawal, pregnancy or postpartum, oral contraceptive use, vaccination, ovulation induction, and other assisted reproductive technology risks. Complete anticoagulation with LMWH is recommended, with enoxaparin dosed at 1 mg/kg every 12 h administered alongside plasma exchange (170, 171). EULAR/ERA-EDTA guidelines recommend high-dose corticosteroids and alternating induction therapy with cyclophosphamide (CTX) or mycophenolic acid derivatives. B-cell targeted therapy, multitarget protocols, plasma exchange or immunoadsorption, and stem cell transplantation are additional reported options (90).

Case studies indicate rituximab improves CAPS symptoms and platelet counts (172). In one CAPS patient with heparin-induced thrombocytopenia, argatroban was substituted for heparin and combined with rituximab, corticosteroids, plasma exchange, and sirolimus. For CAPS secondary to SLE, regimens combining plasma exchange, pulsed methylprednisolone, cyclophosphamide, and anticoagulants have yielded significant improvement (173, 174). Hematopoietic stem cell transplantation (HSCT) is a therapeutic option for SLE-APS patients refractory to standard treatment (175), as demonstrated by reduced clinical symptoms and anti-cardiolipin antibody levels in a refractory APS case with myocardial necrosis unresponsive to immunosuppression (176).

### 4.4 Emerging treatments

#### 4.4.1 Complement-targeted therapeutics

The engineered antibody MBB2ΔCH2 represents a non-complement-fixing anti-β_2_GPI agent derived from a single-chain variable fragment (scFv) that recognizes β_2_GPI across species. This modified construct incorporates the IgG1 hinge, CH2, and CH3 domains to form an scFv-Fc fusion protein. It specifically targets β_2_GPI domain I-mediated thrombosis and fetal loss, thereby mimicking pathogenic antibodies in APS patients. As an innovative therapeutic approach, CH2-deficient antibodies like MBB2ΔCH2 may benefit APS patients refractory to standard therapies. In such refractory cases, a cofactor-independent anticardiolipin-reactive IgG component induces complement-independent monocyte phosphatidylserine (PS) exposure and protein disulfide isomerase (PDI)-dependent tissue factor (TF) activation (177).

#### 4.4.2 Metabolic regulation

##### 4.4.2.1 Cholesterol-binding agents

Statins lower cholesterol and improve endothelial function through potent inhibition of HMG-CoA reductase, reducing oxidative stress and inflammation while modulating immune responses (178). Animal studies demonstrate that simvastatin decreases NETs to reduce oxidative stress, improves neutrophil function, lowers placental resorption rates, and remodels placental blood perfusion, thereby enhancing pregnancy outcomes in OAPS (179). Proteomic analyses reveal altered expressions of Annexin A2, RhoA, and protein disulfide isomerase following fluvastatin intervention. *In vitro* studies indicate that fluvastatin’s inhibition of HMG-CoA reductase involves suppression of isoprenoid synthesis and MAPK activation (180). A clinical investigation involving 42 thrombotic APS patients and 35 healthy donors showed that 20 mg/day fluvastatin for 1 month significantly inhibits monocyte expression of tissue factor, protease-activated receptors 1/2, vascular endothelial growth factor, and Flt-1. Eleftheria Lefkou et al. further found that statin therapy may improve pregnancy outcomes in refractory OAPS patients with preeclampsia or IUGR despite conventional LDA plus LMWH anticoagulation (181).

In addition to statins, methyl-β-cyclodextrin (MβCD) is also an important inhibitor of lipid drafts, which mainly destroys lipid drafts by depleting cholesterol (182, 183). Mβcd can inhibit platelet activation and platelet aggregation by affecting the Ga (i) signaling pathway downstream of the P2Y12 receptor (184). Previous experiments have also demonstrated that MβCD can achieve the effect of anti-atherosclerosis by inhibiting monocyte-endothelial cell adhesion induced by LPS/oxLDL (185). It can serve as an adjunctive inclusion complex of statins, effectively enhancing water solubility and oral bioavailability and reducing toxicity (186, 187). Meanwhile, cyclodextrin (CD) is also used as a pharmacological excipient to improve drug properties, increase drug dissolution rate, and improve drug quality (188, 189).

##### 4.4.2.2 Vitamin D

Vitamin D functions as an anti-inflammatory agent that regulates T and B cells, playing a vital role in immune regulation. Immune cells synthesize active vitamin D metabolites, and vitamin D activation downregulates MHC Class II and co-stimulatory molecules on dendritic cells, thereby reducing T-cell activation. Additionally, activated vitamin D or its analogues inhibit dendritic cell cytokines that promote T helper cell differentiation into Th1 and Th17 cells via IL-23, while also enhancing anti-inflammatory cytokine expression (190).

Previous studies demonstrate that serum 25-hydroxyvitamin D concentrations inversely correlate with SLE disease activity (191, 192). Rohan Willis et al. monitored aPL, 25-hydroxyvitamin D, and proinflammatory cytokines in 312 SLE patients, confirming that low vitamin D levels correlate with elevated inflammatory markers in both SLE and APS. Their findings highlight IL-8’s role in SLE-related obstetric pathology and emphasize the need for active vitamin D monitoring in deficient populations (193). Furthermore, vitamin D deficiency triggers ANA antibody production and is associated with elevated serum IL-23 and IL-17 levels, potentially activating SLE inflammatory mechanisms and exacerbating disease activity (194, 195).

Studies indicate higher vitamin D deficiency prevalence in female SLE patients, correlating with complement consumption and increased disease activity (196, 197). A South Asian phase II study and a Portuguese cohort study establish vitamin D supplementation as safe and effective. Short-term high-dose oral regimens significantly increase vitamin D levels, improve Treg/Th17 ratios, regulate effector T-cell function, and reduce flare risk in stable SLE patients (198).

#### 4.4.3 Abciximab

Abciximab is a chimeric human-mouse monoclonal antibody Fab fragment whose variable region maintains high affinity for the platelet surface GPIIb/IIIa receptor through competitive inhibition. This agent selectively blocks adhesion molecules such as fibrinogen and von Willebrand factor from binding to activated platelet GPIIb/IIIa receptors, thereby preventing thrombosis triggered by vascular injury or atherosclerotic plaque rupture. Additionally, abciximab enhances the fibrinolytic system by inhibiting coagulation factor FXIIIa binding to platelets. Beyond its antiplatelet effects, abciximab cross-reacts with other integrin family members, including macrophage antigen-1 (Mac-1) and the αvβ3 vitronectin receptor; this heteroreceptor binding may confer anti-inflammatory effects via modulation of inflammatory signaling pathways (199).

Clinically, abciximab carries a risk of delayed-onset thrombocytopenia. Existing data indicate a thrombocytopenia incidence of 1-2% after a single initial injection, rising to >10% in patients receiving repeat injections within 30 days (200). Pharmacokinetic studies demonstrate a mean latency period of approximately 9 days for drug-induced thrombocytopenia. Consequently, strict hematological monitoring is recommended during repeated administration to track platelet kinetics (201–203).

#### 4.4.4 Nuclear factor κB and p38 mitogen-activated protein kinase inhibitors

DHMEQ—a weak antibiotic derived from amycolatopsis—suppresses NF-κB activity. It reduces CX3CL1 and CCL5 expression in vascular endothelial cells stimulated by IL-1β and TNF-α across inflammatory and cancer models, although its tissue stability and toxicity require further investigation. The artemisinin analog SM905 inhibits NO and pro-inflammatory cytokine production in LPS-stimulated RAW 264.7 cells, partially via MAPK and NF-κB pathway inhibition (204). SM934 prevents lupus-associated APS by activating Nrf2 and its targets, ameliorating APS-associated lupus nephritis (205). Human umbilical cord mesenchymal stem cell-derived exosomes downregulate inflammatory factors and suppress NF-κB signaling through the miR-146a-5p/TRAF6 axis, ultimately improving trophoblast and placental dysfunction in OAPS mice (206).

## 5 Management

Patients with APS require risk-stratified management. High-risk APS includes individuals with multiple aPL positivity and persistent lupus anticoagulant activity. It also includes those with consistently elevated aPL titers, a history of thrombotic or obstetric APS, or concurrent autoimmune diseases such as SLE. Management should address cardiovascular risk factors, including quitting smoking and strictly controlling blood pressure, lipid levels, and glycemic control. Essential components of APS care include patient education, routine INR monitoring for those on VKAs, perioperative LMWH bridging therapy, and guidance regarding hormonal therapies and lifestyle modifications (226).

### 5.1 Anticoagulation prediction

Anticoagulation is central to thrombotic APS management, balancing thrombosis prevention against bleeding risk. Timely dose adjustment is critical, particularly in patients with severe renal impairment, refractory APS, pregnancy, or triple-positive aPL profiles, which are associated with an increased risk of secondary catastrophic APS. Monitoring strategies incorporate prothrombin time (PT-INR), point-of-care PT-INR testing, aPTT, TT, chromogenic anti-factor Xa assay, and thrombin generation assay.

According to the above indicators and the antibody titer measurement, the risk of thrombosis in aPL can be stratified. The GAPSS is a thrombosis risk assessment system for SLE, incorporating both aPL profiles, particularly aPS/PT antibodies, and cardiovascular risk factors like hyperlipidemia and hypertension (227, 228). This score ranges from 0 to 20 and can be used to predict thrombosis risk and pregnancy complications in SLE patients. Studies show that patients with thrombosis have higher GAPSS than those without thrombosis. In a retrospective cohort study, women with prior aPL-related pregnancy complications had elevated GAPSS. A score ≥16 predicts thrombosis and retains predictive value even without aPS/PT testing (96). These findings indicate GAPSS can effectively predict first thrombotic events in primary APS, but its accuracy requires broader validation (229). Other models that can be used to predict venous thromboembolism risk include the Padua score for hospitalized medical patients and the Caprini score for surgical and medical patients. Additionally, fully evaluating the anticoagulant pharmacodynamics and pharmacokinetics of NOACs is also an important means of monitoring the anticoagulant effect.

The ISTH guidelines recommend full-dose anticoagulation after acute VTE in cancer patients with a high risk of thrombus propagation and a platelet count > 50×10^9^/L. They also recommend platelet transfusion support for thrombocytopenic patients to maintain platelet counts. APS patients may increase platelets through immune regulation to prevent coagulation. Platelet counts should be monitored during the first 14 days and beyond LMWH treatment in patients at intermediate risk of HIT to guide consideration of dose reduction for secondary thromboprophylaxis. NOACs are anticoagulants for >48 h and, like VKA, should be used with caution in thrombocytopenic patients with thrombotic APS. For patients receiving anticoagulation with LMWH, its dosing should be adjusted for APS-associated thrombocytopenia (230).

### 5.2 OAPS management of APS

The strategy for implementing a multidisciplinary approach to preconception counseling for SLE/APS patients is to follow a clinical pathway that guides comprehensive evaluation and management (231). Therefore, based on EULAR/ERA-EDTA/ACR recommendations, multidisciplinary pre-pregnancy counseling and family planning should be implemented for SLE/APS patients to improve adverse pregnancy outcomes (210). Current studies demonstrate that disease activity and serological activity are significantly associated with live birth rates in pregnant women with SLE and APO. These associations are consistent with previous findings. Therefore, accurate risk assessment for live birth is necessary to improve neonatal and maternal outcomes, and SLEDAI is a reliable measurement tool to assess disease activity and to predict preterm birth and pre-eclampsia pregnancies resulting in live births. Compared with the controls, OAPS patients with thrombocytopenia had higher rates of SGA infants (12.12% vs. 31.25%, p = 0.043), preterm birth before 37 weeks (16.16% vs. 43.75%, p = 0.010), and intrauterine fetal death (2.02% vs. 12.50%, p = 0.043). Pre-eclampsia and eclampsia have been associated with adverse pregnancy outcomes (232); women with SLE have a higher risk of eclampsia compared with healthy women; and the infiltration of angiogenesis-associated NETs into placental tissue is enhanced in patients with pre-eclampsia. The International Federation of Gynecology and Obstetrics (FIGO) recommends early pre-eclampsia screening and prevention, including first-trimester screening for early-onset PE and aspirin prophylaxis at a dose of 150 mg per night from 11 weeks to 14 weeks and 6 days of pregnancy until 36 weeks of pregnancy, delivery, or diagnosis of PE (233). During pregnancy, fetal testing, including Doppler ultrasonography and fetal biometry, should be performed to screen for placental insufficiency and SGA infants.

Regarding diagnosis and prevention, recent studies indicate that in aPL-positive pregnant women, whether with or without SLE, anti-phosphatidylserine-prothrombin IgG (aD1) can be an important independent predictor of adverse pregnancy outcomes. There is also a strong association with lupus anticoagulants, and the combination of these two tests can identify patients most likely to develop serious obstetric complications (234). Female fertility may be impaired by disease activity, immunosuppressive drugs, treatment-induced amenorrhea, and renal insufficiency. Cytotoxic drugs reduce fertility, with alkylating agents causing dose-dependent gonadotoxicity through cortical fibrosis, ovarian vascular damage, and ovarian function destruction. Gonadotropin-releasing hormone analogues should be considered before alkylating agent use to preserve fertility (235). APS patients need to use LMWH for anticoagulation during pregnancy, but warfarin is contraindicated. SLE patients should achieve low disease activity pre-pregnancy and take pregnancy-compatible medications, including HCQ, azathioprine, cyclosporin, tacrolimus, and LDA (236, 237). HCQ use during pregnancy improves obstetric complications and live birth rates. Vitamin D deficiency also correlates with adverse pregnancy outcomes; supplementation benefits immune regulation, inflammation prevention, and maternal–fetal health (238). Immunosuppressive therapy requires cervical cancer vigilance with preemptive vaccination. Leflunomide, methotrexate, mycophenolate, and cyclophosphamide are pregnancy-contraindicated.

### 5.3 CAPS management of APS

CAPS primarily causes cell damage through the activation of the complement cascade by aPLs, leading to coagulation and thrombosis, with widespread systemic thrombosis and multiple organ failure as its hallmark features (239). Infection is the most common trigger, followed by surgery, malignancy, pregnancy, anticoagulant discontinuation or inadequate therapy, and SLE. CAPS has a high mortality rate and presents not only with widespread thrombosis but also with fatal systemic inflammation; some patients also develop skin, ocular, and brain involvement (92). The complex multisystem manifestations pose significant diagnostic and therapeutic challenges, complicating treatment planning and anticoagulant application. Thus, multidisciplinary team management is essential. Expert teams, including but not limited to rheumatology, hematology, intensive care, nephrology, obstetrics, and other specialties, collaborate to monitor indicators and adjust therapy (123, 169).

The CAPS Registry (European Antiphospholipid Antibody Forum initiative) indicates that early intervention prevents CAPS based on published cases, registry data, and analyses of cases from Shanghai. Preventive strategies include the use of anti-infective agents, monitoring anticoagulation intensity, and prevention measures (240). Warfarin or LMWH is recommended after assessment of bleeding risk; cyclophosphamide or rituximab is used when CAPS is combined with lupus or other vasculitis (169). Some scholars compared drug combinations in triple therapy, modified triple therapy, and other treatments, finding that the combination of anticoagulation, pulse-dose corticosteroids, and plasma exchange and intravenous immunoglobulin (IVIG) had the highest disease remission rate and was positively correlated with the survival rate of CAPS (241). Plasmapheresis can remove aPLs and cytokines from plasma (173). Perioperative management researchers recorded data from 584 patients and found that eculizumab is recommended for the treatment of refractory CAPS, including thrombotic microangiopathy (152).

### 5.4 pAPS management of APS

The general management of APS in children and adolescents primarily involves the prevention of thrombosis by addressing risk factors, including obesity, arterial hypertension, smoking, and dyslipidemia, along with avoidance of estrogen-containing oral contraceptives and screening for other pre-thrombotic risk factors. Management of mental health issues related to lifestyle and treatment adherence is also recommended (242). The major manifestations of pAPS include large vessel thrombosis and thrombotic microangiopathy (174). Acute thrombosis treatment in pAPS follows adult primary APS protocols. For childhood-onset systemic lupus erythematosus (cSLE) and pAPS, care should address vaccination, exercise training, medication use, and complication monitoring. For APS patients taking anticoagulants, participation in contact sports should be avoided to prevent acute local or systemic bleeding. Nevertheless, exercise training is potentially beneficial for childhood rheumatic disorders such as cSLE and pAPS (243). Studies demonstrate that a 12-week supervised aerobic training program is safe and beneficial in increasing aerobic endurance and physical function in cSLE patients with pAPS. In addition, pAPS patients on long-term anticoagulant medication need regular monitoring of bone health to prevent osteoporosis. Vaccination serves as an effective thrombosis prevention strategy. cSLE patients, particularly those immunosuppressed, should receive inactivated vaccines; live attenuated vaccines are generally contraindicated (244). Additionally, inherited complement deficiency represents a key risk factor for cSLE and pAPS, with such individuals often developing lupus early. Screening cSLE patients for complement defects is therefore valuable. One study reported two cases of coagulation abnormalities after renal biopsy in children with SLE and APS, resulting in a mortality risk (245). Consequently, coagulation risk must be carefully monitored to ensure treatment safety and efficacy.

Daily management for cSLE patients requires sun protection against mucocutaneous damage. Case analyses reveal that pediatric APS patients with cSLE may present diverse ocular symptoms such as reticular rash and optic nerve disorders (246). Concurrently, common SLE medications, such as HCQ, may adversely affect eye health; thus, annual eye examinations are recommended. At the same time, medications such as common heparin, LMWH, and VKA can impair bone density and metabolism. Therefore, children and adolescents with APS should maintain adequate calcium and vitamin D intake for bone health.

### 5.5 Perioperative management of APS

Perioperative management of APS requires careful balancing of thrombotic risk and bleeding complications. Abrupt withdrawal of oral warfarin therapy may induce transient hypercoagulability through increased fibrin and thrombin generation. Guidelines recommend heparin bridging therapy for high-thrombotic-risk patients undergoing invasive procedures, as continuing therapeutic anticoagulation reduces thrombotic TMA risk (240). However, perioperative anticoagulation increases bleeding risk despite mitigating TMA risk. Furthermore, abrupt cessation of anticoagulants preoperatively can elevate fibrin and thrombin production, causing transient hypercoagulability (247). Both the American College of Chest Physicians (ACCP) and the UK National Institute for Health and Care Excellence (NICE) advise heparin bridging therapy for patients discontinuing warfarin to reduce surgical hemorrhage risk who also face significant thrombotic risk (248).

Barbour et al. reported a case of a patient with SLE and APS who underwent kidney transplantation. Preoperatively managed with LMWH and warfarin, the patient developed acute allograft dysfunction due to TMA despite continuous preoperative anticoagulation (249). This highlights that surgical interventions can trigger ischemia–reperfusion injury, complement activation, and CAPS through coagulation cascade activation. Uninterrupted UPH bridging therapy is recommended for triple-positive APS patients, particularly those with secondary APS, to minimize anticoagulation gaps.

Based on case studies, oral warfarin therapy should be reinstated postoperatively on the night of surgery, with extended thromboprophylaxis using low-dose heparin or LMWH for patients on long-term warfarin therapy, barring surgical contraindications. Intermittent pneumatic compression devices and graduated compression stockings help reduce DVT incidence by decreasing venous stasis (250).

## 6 Conclusions

The core pathophysiology of antiphospholipid antibody syndrome lies in the interaction between circulating aPL and the β2GPI. This interaction not only directly activates the procoagulant state of vascular endothelial cells and platelets but also simultaneously triggers the complement cascade and inflammatory pathways, ultimately leading to a widespread thrombotic tendency or obstetric complications. Although diagnosis relies on clinical criteria and persistent laboratory positivity for aPL, current standards still fail to cover all clinical phenotypes. Moreover, the complexity of the antibody spectrum and the risk stratification of thrombotic/obstetric events remain major challenges.

Looking ahead, APS research and management must advance in multiple dimensions; the highest priority is developing more precise disease classification and risk stratification strategies through integrating comprehensive antibody profiles, genetic backgrounds, and biomarkers such as complement activation to achieve individualized risk assessment. Subsequently, based on a deeper understanding of the pathological mechanisms, rigorously designed clinical trials targeting specific pathways are urgently needed to provide new therapeutic options for patients refractory to or with contraindications for traditional anticoagulation or antiplatelet therapy. Furthermore, long-term management should specifically address comorbidities, optimize pregnancy protocols, and mitigate bleeding risks associated with anticoagulation. Ultimately, through advancing our understanding of APS’s complex pathological network and developing precision medicine approaches, we can more effectively combat this autoimmune disease that causes thrombosis and pregnancy loss, significantly improving patients’ quality of life and long-term prognosis.

## Glossary

APS: antiphospholipid syndrome
SLE: systemic lupus erythematosus
aPLs: antiphospholipid antibodies
β_2_GPI: beta-2 glycoprotein I
LA: lupus anticoagulant
aCL: anti-cardiolipin antibodies
anti-β_2_GPI: anti-beta-2 glycoprotein I antibodies
TLR: Toll-like receptor
C5aR: C5a receptor
MyD88: myeloid differentiation primary response 88
NF-κB: nuclear factor kappa-light-chain-enhancer of activated B cells
TNF-α: tumor necrosis factor-alpha
IL: interleukin
ROS: reactive oxygen species
TF: tissue factor
NETosis: neutrophil extracellular trap formation
PI3K: phosphoinositide 3-kinase
mTOR: mammalian target of rapamycin
E-selectin: E-selectin
VCAM-1: vascular cell adhesion molecule-1
ICAM-1: intercellular adhesion molecule-1
TLR: Toll-like receptor
LRP: low-density lipoprotein receptor-related protein
IFN-γ: interferon-gamma
BAFF: B-cell activating factor
C5a: complement component 5a
MAC: membrane attack complex
TM: thrombomodulin
APC: activated protein C
ATIII: antithrombin III
NO: nitric oxide
PGI2: prostacyclin
TFPI: tissue factor pathway inhibitor
u-PA: urokinase-type plasminogen activator
t-PA: tissue-type plasminogen activator
PAI-1: plasminogen activator inhibitor-1
FDP: fibrin degradation products
dRVVT: diluted Russell’s viper venom time
aPTT: activated partial thromboplastin Tim
aPS/PT: anti-phosphatidylserine/prothrombin antibody
PS: phosphatidylserine
Annexin II/V: Annexin II/V
anti-ANXA5: anti-Annexin A5 antibodies
A2: Annexin II
PADI4: peptidyl arginine deiminase 4
ELANE: elastase, neutrophil expressed
MPO: myeloperoxidase
NADPH: nicotinamide adenine dinucleotide phosphate
SN-APS: seronegative antiphospholipid syndrome
PH: pulmonary hypertension
PE: pulmonary embolism
IPAH: idiopathic pulmonary arterial hypertension
PAH: pulmonary arterial hypertension
CAD: coronary artery disease
APSN: antiphospholipid syndrome nephropathy
TMA: thrombotic microangiopathy
CAPS: catastrophic antiphospholipid syndrome
pAPS: pediatric antiphospholipid syndrome
OAPS: obstetric antiphospholipid syndrome
IUGR: intrauterine growth restriction
HELLP: hemolysis, elevated liver enzymes, low platelets
DOACs: direct oral anticoagulants
LMWH: low molecular weight heparin
UFH: unfractionated heparin
fondaparinux: fondaparinux
HCQ: hydroxychloroquine
LDA: low-dose aspirin
VKA: vitamin K antagonist
INR: international normalized ratio
GAPSS: Global Antiphospholipid Syndrome Score
IVIg: intravenous immunoglobulin
CTX: cyclophosphamide
eGFR: estimated glomerular filtration rate
LDH: lactate dehydrogenase
SLEDAI: Systemic Lupus Erythematosus Disease Activity Index
FIGO: International Federation of Gynecology and Obstetrics
ANA: antinuclear antibodies
ACCP: American College of Chest Physicians
NICE: National Institute for Health and Care Excellence
DVT: deep vein thrombosis
TIA: transient ischemic attack
VEGF: vascular endothelial growth factor
EVs: extracellular vesicles
Th1: T helper 1 cells
Th17: T helper 17 cells
Treg: regulatory T cells
HLA: human leukocyte antigen
SNP: single-nucleotide polymorphism
LDG: low-density granulocyte
PMN-MDSC: polymorphonuclear myeloid-derived suppressor cells
MHC: major histocompatibility complex
BCMA: B-cell maturation antigen
STAT4: signal transducer and activator of transcription 4
NETs: neutrophil extracellular traps
BLyS: B-lymphocyte stimulator
TRAF6: TNF receptor-associated factor 6
MβCD: methyl-β-cyclodextrin
DKK1: Dickkopf-related protein 1
IRAK: interleukin-1 receptor-associated kinase
TRIF: TIR-domain-containing adapter-inducing interferon-β
MAPK: mitogen-activated protein kinase
MEK-1/ERK: mitogen-activated protein kinase kinase 1/extracellular signal-regulated
PAR: protease-activated receptor
FXa: activated factor X
C3a: complement component 3a
C5b-9: complement membrane attack complex
TIRAP: TIR-domain-containing adapter protein
oxLDL: oxidized low-density lipoprotein
scFv: single-chain variable fragment
HMG-CoA: 3-hydroxy-3-methylglutaryl-coenzyme A
PDI: protein disulfide isomerase
cSLE: childhood-onset systemic lupus.
