# Correction: Novel advances on pathophysiological mechanisms, clinical manifestations, and treatment of antiphospholipid syndrome

**DOI:** 10.3389/fimmu.2025.1711668

**Published:** 2025-10-08

**Authors:** Qing-Nan Zhu, Xiang-Bo Qi, Shu-Wei Ren, Yu-Ye Li, Ze-Wen Yan, Yu Sun, Yan Shi, Qing-Si Wen, Mao-Mao Wu, Da-Peng Wang

**Affiliations:** ^1^ Department of Nephrology, The First Affiliated Hospital, Dalian Medical University, Dalian, China; ^2^ Institute of Integrative Medicine, Dalian Medical University, Dalian, China

**Keywords:** antiphospholipid syndrome, systemic lupus erythematosus, thromboinflammation, thrombosis, anticoagulants

Affiliation “Department of Nephrology, The First Affiliated Hospital, Dalian Medical University, Dalian, China” was omitted for author Qing-Nan Zhu. This affiliation has now been added for author Qing-Nan Zhu. As such, affiliation “Institute of Integrative Medicine, Dalian Medical University, Dalian, China” previously listed as affiliation 1 is now affiliation 2 and the author list has been updated accordingly.

In the published article, there was a mistake in the Figure order. The image labeled as “**Figure 1**” should be “**Figure 2**” and the image labeled as “**Figure 2**” should be “**Figure 1**”. The order has now been corrected.

**Figure 1:**
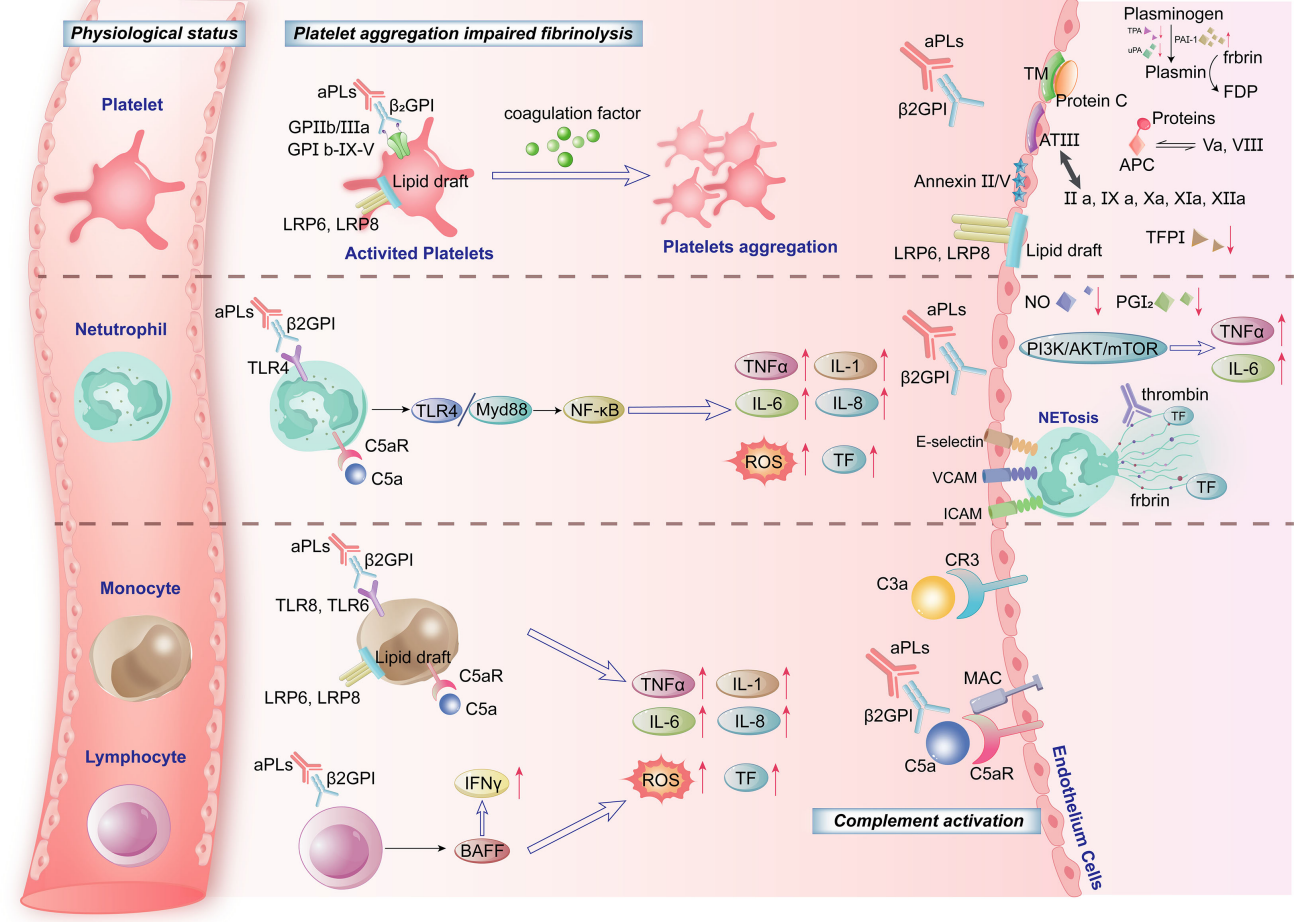
Pathogenesis of antiphospholipid syndrome (APS): cellular mechanisms.

**Figure 2:**
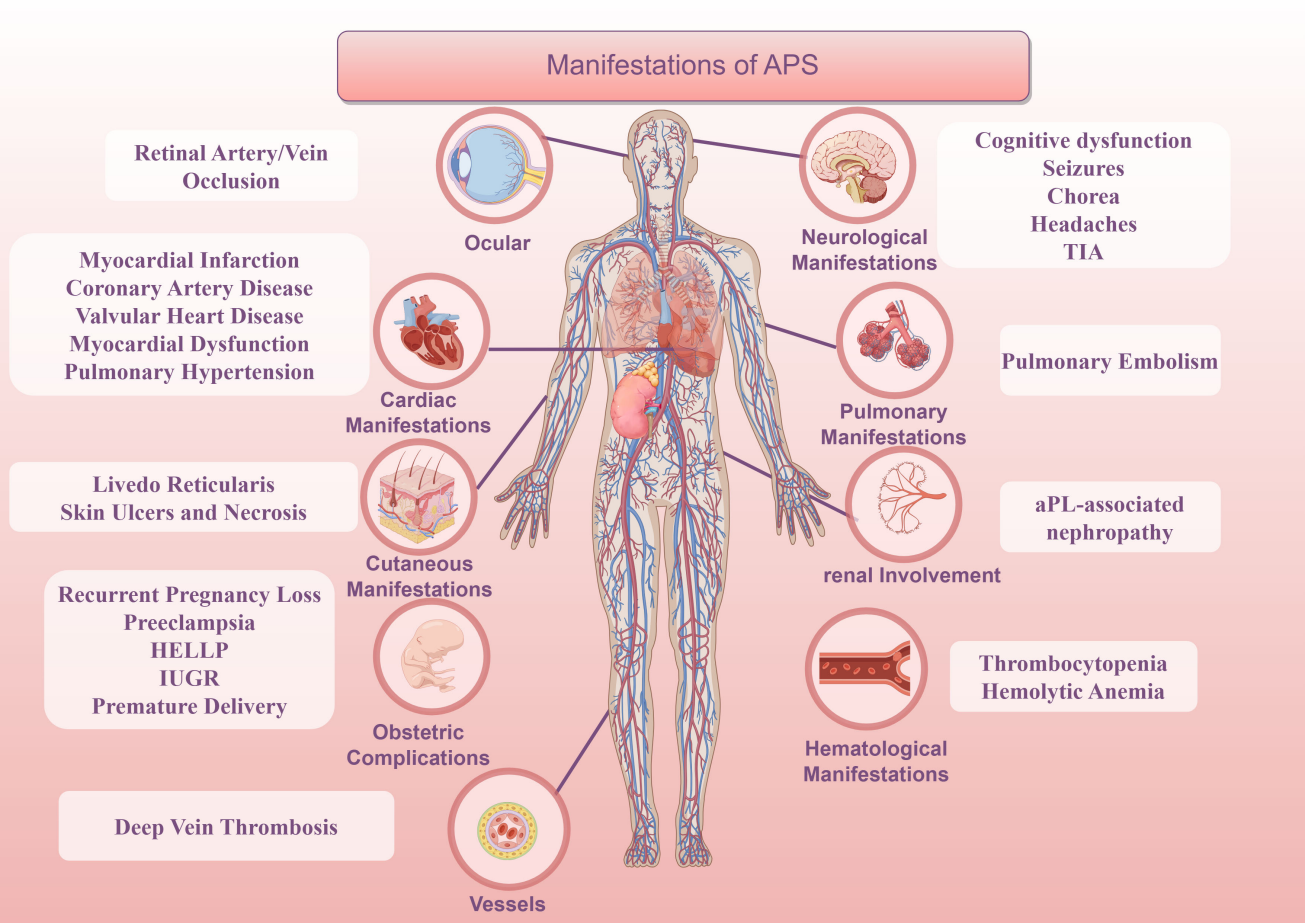
Manifestation of APS.

The original version of this article has been updated.

